# Craniux: A LabVIEW-Based Modular Software Framework for Brain-Machine Interface Research

**DOI:** 10.1155/2011/363565

**Published:** 2011-04-07

**Authors:** Alan D. Degenhart, John W. Kelly, Robin C. Ashmore, Jennifer L. Collinger, Elizabeth C. Tyler-Kabara, Douglas J. Weber, Wei Wang

**Affiliations:** ^1^Department of Bioengineering, University of Pittsburgh, Pittsburgh, PA 15219, USA; ^2^Department of Electrical and Computer Engineering, Carnegie Mellon University, Pittsburgh, PA 15213, USA; ^3^Department of Physical Medicine and Rehabilitation, University of Pittsburgh, Pittsburgh, PA 15213, USA; ^4^Department of Veterans Affairs, Human Engineering Research Laboratories, Pittsburgh, PA 15206, USA; ^5^Department of Neurological Surgery, University of Pittsburgh, Pittsburgh, PA 15213, USA

## Abstract

This paper presents “Craniux,” an open-access, open-source software framework for brain-machine interface (BMI) research. Developed in LabVIEW, a high-level graphical programming environment, Craniux offers both out-of-the-box functionality and a modular BMI software framework that is easily extendable. Specifically, it allows researchers to take advantage of multiple features inherent to the LabVIEW environment for on-the-fly data visualization, parallel processing, multithreading, and data saving. This paper introduces the basic features and system architecture of Craniux and describes the validation of the system under real-time BMI operation using simulated and real electrocorticographic (ECoG) signals. Our results indicate that Craniux is able to operate consistently in real time, enabling a seamless work flow to achieve brain control of cursor movement. The Craniux software framework is made available to the scientific research community to provide a LabVIEW-based BMI software platform for future BMI research and development.

## 1. Introduction

Brain-machine interface (BMI) technology aims to establish a direct link for transmitting information between the brain and external devices. It offers a rich and natural assistive device control interface for individuals with disabilities [1, 2] and is a rapidly-progressing, extremely active research area in the field of neuroscience and neural engineering. Various neural signal modalities, including electroencephalography (EEG) [3], magnetoencephalography (MEG) [4], electrocorticography (ECoG) [5], intracortical local field potentials (LFPs) [6], and neuronal firing rates [7–9], have been used for BMI research. Regardless of the input modality, all BMI systems require an essential suite of software capable of acquiring neural signals continuously and converting them in real time or near real time into specific BMI control commands for an external device, such as a prosthetic hand, in order to accomplish a specific task.

To conduct innovative and unique BMI studies, researchers very often need to implement new signal processing techniques, neural decoding algorithms, or experimental paradigms in a BMI software package. Given the rapid progression of the field, it is desirable to reduce the time it takes from the conception of a new idea to software implementation, data collection, and data analysis. However, the increasing complexity of BMI systems has made this problematic. For example, sophisticated neural decoding algorithms previously studied in offline analysis are now being investigated for real-time BMI control [10]. Additionally, more advanced external devices are being controlled by BMI systems, such as the dexterous prosthetic arm and hand system developed by the Revolutionizing Prosthetics project [11, 12]. These advancements call for an open-source software framework that enables BMI researchers to better focus on the essential engineering and scientific questions they are investigating and to develop advanced BMI features more efficiently. This framework should be able to manage the basic software operations common to many BMI studies and should be easily extendable in a high-level programming environment that offers the ease and flexibility for programming new BMI modules.

One successful open-source general purpose BMI software package is BCI2000, a modular C++-based system for neural signal acquisition, data saving, stimulus presentation, and more [13], which has been widely distributed among academic institutions and used in numerous research studies [14]. Source code as well as binary executable files are freely available for download, allowing end users to either use the software as is or modify it to suit their own needs. One of the greatest advantages of BCI2000 is its modular, lightweight, and portable design, making it extremely popular and successful in the BMI research community. Recently, the BCPy2000 open-source framework [15] has been made available as a user contribution package to BCI2000. This framework follows the same system architecture as BCI2000, but it allows BMI researchers to develop new modules in Python, a high-level language that greatly reduces software programming complexity for fast prototyping of new BMI software.

This paper presents an open-source open-access real-time BMI software framework inspired by BCI2000 termed “Craniux,” developed using LabVIEW (National Instruments, Inc.), a high-level multiplatform graphical development environment. Craniux implements a core framework for BMI operation, including modular architecture, network communications between modules, data flow control, data visualization, data storage, and graphical user interfaces. Craniux offers a unique set of advantages that can greatly facilitate BMI software development and research. First, it enables BMI researchers to develop and share new BMI modules in the LabVIEW development environment and take full advantage of many features inherent to this environment, such as

- high-level graphical programming for fast development and run-time debugging,
- a rich set of data visualization options and graphical user interface elements,
- ease of multithreading and parallel processing programming, including automatic parallelism and multicore processor support,
- a large number of high-quality LabVIEW function libraries for signal processing and stream-lined integration with a wide range of engineering hardware (e.g., national instruments controller cards),
- reuse and sharing of custom-made LabVIEW modules as sub-VI (virtual instrument) blocks.

Second, facilitated by the above-mentioned LabVIEW features, we have further implemented functionality critical for BMI research

- Real-time operation in which the system is capable of acquiring a block of neural data, processing this data, and generating an output before the next block of data is received [13, 16].
- Online neural decoder training capability accomplished through data sharing between real-time operations and parallel decoder training.
- “On-the-fly” data visualization and online experiment parameter control.
- Deterministic control of system execution, including parameter updates and display of visualization data.
- Streaming and storage of raw neural data, various intermediate processing data, and experimental parameters to disk for offline analysis.
- Distribution of BMI modules across computers using well-defined generic network communication protocols optimized for data transmission between software modules.

Finally, Craniux has been developed to be a lightweight, extendable, and portable software framework. Its modular architecture, well-defined user interfaces, and generic network communication protocol make it very easy to maintain and develop BMI engines. The existing engines and standard template engines provide a starting point for new engine development.

In the following sections, we will first introduce the basic system architecture of the Craniux software. We will then provide system performance testing results based on both simulated and real experimental data. The last section will further discuss the uniqueness of this software framework as compared to other existing BMI software tools, its advantages and limitations, and future directions.

## 2. System Architecture

 The Craniux software package has been designed to be a highly modularized system, capable of operating across both a distributed network of computers and on a single computer. To accomplish this, and to make data transfer between engines as reliable as possible, all data communication is conducted using the TCP/IP protocol. Data saving is implemented using the LabVIEW TDMS (Technical Data Management Streaming) framework [17], ensuring all system data are streamed to disk as quickly as possible in order to maximize system performance. The following sections describe the system framework, engine execution, GUI operation, communication protocols, and data saving operation in further detail.

### 2.1. Distributed Engine Framework

**Figure 1:**
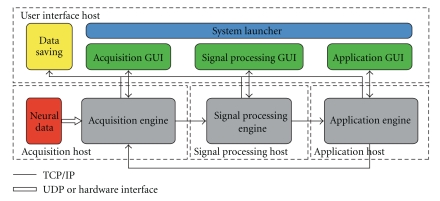
*Craniux system framework.* The Craniux system is comprised of the acquisition, signal processing, and application engines, their associated GUIs, the system launcher, and the data saving manager. Engines and user interface elements are spread across four network hosts: the acquisition host, the signal processing host, the application host, and the user interface host, though the same computer may serve as multiple hosts. Network communication between system engines, as well as communication between engines and GUIs, is performed using the TCP/IP protocol. A block of neural data enters the system through the ACquisition engine, which sends preprocessed data to the Signal processing engine. The signal processing engine generates a control signal, which is then sent to the application engine. The application engine then communicates any relevant application-specific data (e.g., target information used for neural decoder training) back to the acquisition engine, which reads the next block of neural data. Bidirectional data transfer occurs between engine-specific GUIs and their associated engines, with system parameters transferred from the GUI to the engine and visualization data transferred from the engine to the GUI. Finally, the system launcher is responsible for loading the desired engines, tracking general experimental parameters, and experimental control.

Figure 1 depicts the design of the Craniux system. Inspired by the BCI2000 framework, this system consists of five distinct components: the system launcher, acquisition engine, signal processing engine, application engine, and data saving manager and may be distributed across as many as four computers. Furthermore, each engine has an associated graphical user interface (GUI), through which the user interacts with the engine. The main system components perform the following functions.

**System Launcher**

The system launcher is the initial interface the user is presented with when running the software and allows the user to specify system-level parameters at runtime. It is here that the specific engines, their network locations, and high-level experimental parameters (e.g., subject ID, date, investigators, and session number) are specified. Additionally, the system launcher controls the start and stop of execution though it itself is not a part of the real-time operation of the system.

**Acquisition Engine**

Acquisition engines are responsible for the acquisition and initial preprocessing (e.g., spectral estimation) of neural data from some signal source such as an amplifier or user datagram protocol (UDP) connection.

**Signal Processing Engine**

Signal processing engines receive data from the acquisition engine and are responsible for the processing of this data, such as the generation of a control signal.

**Application Engine**

Application engines receive data from the signal processing engine and are responsible for the control of interaction between the subject and the BMI.

**Data Saving Manager**

The data saving manager is responsible for the saving of Craniux data and receives input from the acquisition, signal processing, and application engines.

In order to ensure sequential processing of data through each engine, system execution proceeds from the acquisition engine to the signal processing engine, then to the application engine, and finally from the application engine back to the acquisition engine; only one of each type of engine may be running at a given time. This cyclical data flow guarantees that each block of data received by the acquisition engine is processed and a system output is generated before processing of the next block of data begins.

At any point in operation, the system may be suspended and any of the engines replaced with another of the same type, preserving the state of those engines that remain running. This is desirable for BMI operation, as system parameters such as neural decoder weights obtained during operation with a specific application (e.g., a center-out computer cursor task) may be retained and immediately used for a new application (e.g., the control of a robotic arm).  Table 1 provides a list of the current engines available in the Craniux system.

### 2.2. Engine Execution

**Figure 2:**
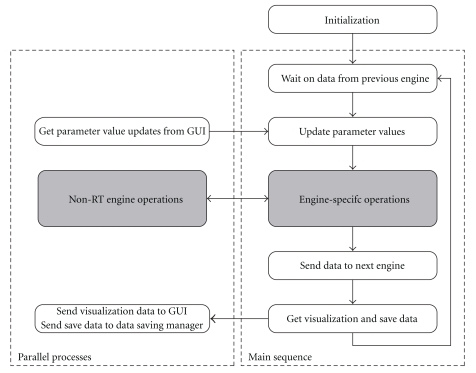
*Engine execution.* After initialization, each engine proceeds into the main sequence loop, in which core engine processes are executed sequentially. Data are first received from the previous engine in the signal chain, and any parameter value changes received from the engine's GUI are updated locally. The system next performs any actions specific to the individual engine (e.g., calculation of a control signal or updating of a display), and sends the results of these actions to the next engine in the signal chain. Current values of any data items to be visualized are placed in a queue, and data is sent to the data saving manager. The engine then proceeds to the beginning of the main sequence loop to await the arrival of the next input. Parallel to the main sequence loop are any parallel processes designed to operate asynchronously. These processes will always include receiving parameter value updates from the GUI and sending visualization data to the GUI, and may include individual engine-specific operation such as decoder training or monitoring for events. Shaded blocks represent those areas to be modified by the developer during the creation of new engines, while white blocks represent sections of code providing core functionality.

 Each engine in the system operates in a basic sequence, first receiving data from a previous engine, processing the received data, and sending the relevant results of that processing to the next engine in the signal chain.  Figure 2 outlines the basic flow of the execution of an individual engine. Engine execution first begins with the initialization of all parameters, including the loading of user-specified parameter files, and the identification of those engine-specific parameters to be saved. From here, execution proceeds into the main sequence of the engine, where the engine (1) waits for data from the previous engine in the signal chain, (2) processes the received data (or performs some other action), and (3) sends the results of this processing to the next engine in the signal chain. Execution then proceeds back to (1), where the engine waits for the next block of input data. Operating in parallel to this main sequence are a number of additional threads, such as data saving, engine-specific processes not capable of or not requiring real-time operation (e.g., neural decoder training), and communication with the engine's GUI. A detailed description of engine execution, including the enforcement of deterministic execution within engine components, is provided in the supplemental materials (see supplementary materials available online at doi:10.1155/2011/363565). 

### 2.3. Graphical User Interface (GUI) Elements

 The GUI for each engine is responsible for both on-the-fly control of engine-specific parameters as well as the visualization of engine-specific data. Permitting on-the-fly control is essential to successful BMI operation, as during real-time closed-loop BMI operation it is often necessary to dynamically adjust parameters such as the computer assist level or computer cursor speed [8]. As opposed to a traditional graphical user interface, which simply serves as a front end user interface for a LabVIEW application, GUIs in the Craniux system exist as stand-alone applications. It is through these applications that the user interacts with each engine. GUIs and their associated engines maintain reciprocal two-way communication; parameter value change events are monitored by the GUI and transmitted to its associated engine via TCP/IP, while data to be visualized is transmitted from the engine to the GUI. It should be noted that parameter value changes are instantaneously transmitted from the GUI to the engine and are accessed by the engine at the beginning of its main sequence in order to ensure the consistency of all parameter values throughout the processing of a single block of data. Parameter value changes are also index stamped and saved to disk, allowing the complete reconstruction or replay of the full system state during offline analysis. In order to allow for data visualization on the fly, all data elements are transmitted from the engine to the GUI, providing the experimenter with the most accurate representation of the state of the engine. This occurs in parallel with the real-time main sequence execution, so that this communication does not interfere with the timing of the execution of the main sequence of the engine.

### 2.4. Communication between Components

 Communication between Craniux components utilizes self-establishing and self-repairing network connections that provide efficient, reliable data flow robust to any data type or combination of variables that is sent over them. For communication between engines, these connections take the form of a ring that maintains data flow and controls program execution. For communication between engines and their GUIs or the data saving manager, a single TCP connection is established. When creating new engines, this ring requires no input and single connections only require the developer to provide a network host name. The only user input necessary is the IP address of each engine, which is specified on the system Launcher. Available ports are automatically selected for each connection.

All network connections use the TCP protocol. TCP was chosen over UDP because its superior reliability is important in a ring structure responsible for the control of program execution; a dropped packet between engines would break the ring and leave each engine waiting for data that will never arrive. It is also important to note that Nagle's algorithm [21] was disabled for all connections used in the Craniux system. The Nagle algorithm attempts to reduce TCP packet overhead and bandwidth usage by intentionally delaying transmission, so that multiple packets can be combined before being sent. Here, the latency introduced by this algorithm is unacceptable, and bandwidth usage is not a concern. The concept behind Nagle's algorithm is retained in our system; however, as all data to be sent simultaneously is combined into a single packet before transmission.

To send variables over the network, the developer must only create a list of the variable names on the sending side of the connection. No information on variable type or size is needed. The provided variable names are packed together with their values into a single variable of LabVIEW's “variant” data type, which is then sent over the network. On the receiving side of the connection, the data is read and parsed into the correct values, which are written to those existing variables on the receiving side with the same name and data type as the sent variables. Additional information on the transfer of information between components has been provided in the supplemental materials.

### 2.5. Data Saving

 The Craniux framework for saving data is a reliable process that minimizes latency introduced by saving and creates highly accessible data. Data saving is conducted by an independent data saving manager, which receives data from all engines. This data is initially saved in LabVIEW's TDMS format, which was specifically created for quickly and continuously streaming large amounts of data to the hard drive to help eliminate data-saving bottlenecks in speed normally introduced by slow writes to disk [17]. When saving data, a packet containing all the variable values to be saved and the data packet index is placed into a first-in-first-out (FIFO) buffer. Parallel to the main execution of Craniux, these packets are removed from the buffer and sent to the data saving manager, located on the user interface host, via the communications framework described in Section 2.4. Upon receiving a packet, the data saving manager streams the data to a TDMS file. When creating new engines, it is only necessary to provide a list of variable names to be saved; these items will be automatically identified and their values saved accordingly.

A single TDMS file is saved for each experimental run; stopping or suspending system execution closes all references to the current data file. Within each data file, data saved by each engine is separated into two groups: sampled variables and controls. Sampled variables are data sampled continuously at each update of the BMI system, such as cursor position during a brain-controlled cursor movement task. As controls are normally parameter settings that are infrequently updated (e.g., the number of targets), these values are only saved when changed. The current data packet number is included in every save operation, so that the experiment can be reconstructed afterwards with the data properly aligned in time. A separate LabVIEW VI has been created to convert Craniux TDMS files into the MATLAB (Mathworks, Inc.) MAT format. These MAT files contain structures for each engine paired with each data type (sampled variables and controls). The saved values for each variable are stored in an array, with the data packet number array providing the time index for each element. Array variables are stored as cell arrays, allowing them to be aligned with their associated data packet numbers and enabling the data structure to handle dynamic changing of array sizes during a BMI session.

## 3. System Validation

### 3.1. Closed-Loop Cursor Movement Control Using Simulated ECoG Signals

**Figure 3:**
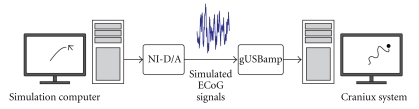
*Simulated ECoG experimental setup.* Experimenter-controlled mouse position on the simulation computer modulates the high-gamma power of simulated directionally tuned ECoG signals. These signals are output at 2400 Hz using a National Instruments D/A card and are read into the Craniux system using the g.USBamp amplification system and BCI2000. The Craniux system then decodes the desired cursor position from the simulated signals using the population vector algorithm.

 The experimental setup used for validation of the Craniux system is shown in Figure 3. An electrocorticographic (ECoG) signal simulator in which experimenter-controlled mouse cursor movement was used to modulate the high gamma-band activity of a number of synthetic signals. This simulator is capable of generating 32 channels of analog signals with directionally tuned high-gamma band (70–120 Hz) activity emulating ECoG signals recorded from human subjects [2, 22] according to the following [6, 18],

(1) $$S=S_{1}+d\cos\;\;\left(\theta \right)S_{2},$$

where *S* is a single simulated directionally modulated ECoG signal, *S*_1_ is a pink noise signal with a 1/frequency power falloff [23]. *S*_2_ is a second pink noise signal band-pass filtered between 70 and 120 Hz, *d* controls the depth of modulation of the high-gamma band, and *θ* is the angle between the preferred direction of the simulated ECoG signal and the vector pointing from the center of the computer screen to the current mouse cursor position on the computer screen. The preferred directions of the 32 simulated signals were uniformly distributed over two-dimensional (2D) space. Simulated signals were generated at 2400 Hz using a National Instruments NI PCI-6723 32 channel analog output board on a simulation computer (Windows XP x86 operating system, AMD Athlon 64 FX-62 Dual Core CPU @ 2.81 GHz, 3.5 GB RAM, NVIDIA GeForce 7900 GS video card) and then stepped down to match the amplitude of typical ECoG signals recorded from human subjects.

Simulated ECoG signals were then sampled at 1200 Hz using the g.USBamp amplification system (Guger Technologies, OEG) on a separate computer (Windows XP x86 operating system, Intel Core i7 CPU 920 @ 2.67 GHz, 2.49 GB RAM, 2 NVIDIA GeForce 9800 GT video cards) and sent to the Craniux system as binary UDP packets using a simplified version of the BCI2000 software package. BCI2000 was used in this case due to its reliability and efficiency in interfacing with the g.USBamp amplification system. These raw time-domain signals entering the Craniux system were first converted into the frequency domain using LabVIEW's built-in autoregressive (AR) spectral estimation function (10 Hz bins, 500 ms window) in the read UDP binary acquisition engine and then passed to the population vector signal processing engine. Here, signals were normalized to pseudo-Z-scores based on the following [24, 25],

(2) $$\begin{array}{c} f_{\text{ norm},i,j}=\frac{f_{i,j}-{\bar{f}}_{i,j}}{\sigma_{i,j}}, \end{array}$$

where *f*_norm,*i*,*j*_, *f*_*i*,*j*_, and ${\bar{f}}_{i,j}$ are the normalized, raw, and mean power of the *i*th channel and *j*th frequency band, respectively, and *σ*_*i*,*j*_ is the standard deviation of the raw band power of the *i*th channel and *j*th frequency band. Mean and standard deviation values were calculated based on data collected during a baseline condition in which the computer cursor on the simulation computer remained in the center of the screen (i.e., no modulation of high-gamma band activity).

The brain control task used was a typical 2D center-out design, with the movement direction of a cursor controlled by multiple ECoG signal features across 32 channels according to the population vector algorithm [18]

(3) $$\begin{array}{c} f_{i}=b_{0,i}+b_{x,i}m_{x}+b_{y,i}m_{y},\\ P=\overset{N}{\underset{i}{\sum }}\left(d_{i}-b_{0,i}\right)C_{i}, \end{array}$$

where *f*_*i*_ is the activity of individual feature *i*, *m*_*x*_, and *m*_*y*_ are the desired movement in the *x* and *y* direction, *b*_0,*i*_, *b*_*x*,*i*_, and *b*_*y*,*i*_ are coefficients found using linear regression relating desired movement to the activity of feature *i*, *P*_*i*_ is the trajectory vector predicted by the activity of feature *i*, *d*_*i*_ is the instantaneous activity of feature *i*, and **C**_*i*_ = [*b*_*x*,*i*_ *b*_*y*,*i*_]/(*b*_*x*,*i*_^2^+*b*_*y*,*i*_^2^)^1/2^ is a vector representing the preferred direction of feature *i*.

The standard workflow used to achieve ECoG-controlled 2D cursor movement with the Craniux framework is described below. Though simulated ECoG signals were used here to validate the system, this workflow will be similar for real neural signals.

**Figure 4:**
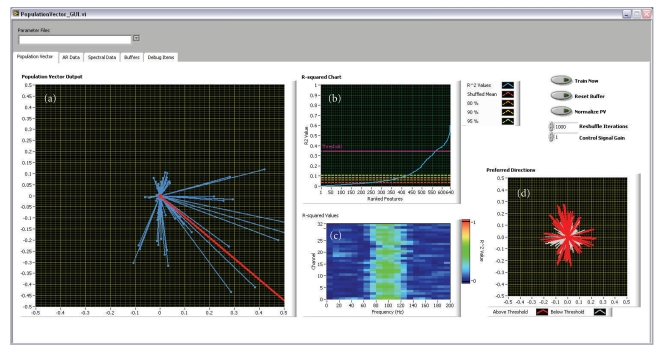
*Craniux system screenshot during population vector-based control.* (a) Plot of the instantaneous activity of each feature used for cursor control along its preferred direction (blue) and the resultant population vector (red). (b) *R* ^2^ value plot indicating the distribution of *R* ^2^ values obtained during population vector training (blue) compared to the mean, 80th, 90th, and 95th percentile *R* ^2^ values obtained after training on 1000 iterations of target-shuffled data (red, dark orange, light orange, and yellow lines). The threshold above which features are chosen for use in the decoder is shown by the pink line. (c) *R* ^2^ values obtained during population vector training arranged by channel and frequency band. Note that the 70–120 Hz frequency band features show high *R* ^2^ values across all channels, consistent with the method used to generate the simulated ECoG signals. (d) The preferred direction distribution of all features. Red lines correspond to those features with *R* ^2^ values above the user-determined threshold, while white lines are those features falling below the threshold.

1. *Collection of Baseline Data.* Once the Craniux system is started, approximately 3 minutes of baseline data is collected, from which the Craniux system will calculate feature mean and standard deviation values. These will then be used in the calculation of pseudo-Z-scores for all ECoG signal features in real time.
2. *Collection of Training Data for the Neural Decoder.* During this period, the experimenter will use the ECoG signal simulator to generate modulated ECoG signals based on the target position (i.e., desired cursor movement direction). The ECoG data along with target position are automatically buffered by Craniux for neural decoder training.
3. *Training of the Neural Decoder.* During this period, the buffered data is used to train the neural decoder. A multiple linear regression procedure is used to determine the degree of directional tuning and preferred direction for each ECoG signal feature as mentioned above [26, 27]. The resulting *R*-squared values and preferred directions are displayed by the population vector GUI, allowing experimenters to visualize the results on the fly and interactively select a subset of directionally tuned ECoG signal features for brain control.
4. *Real-Time Brain Control.* Activities of ECoG signal features selected during step (3) are then used to generate the population vector, a 2D velocity control signal that drives the cursor.  Figure 4 shows the population vector GUI during closed-loop brain control, illustrating the user interface elements provided to the user during this process.

**Figure 5:**
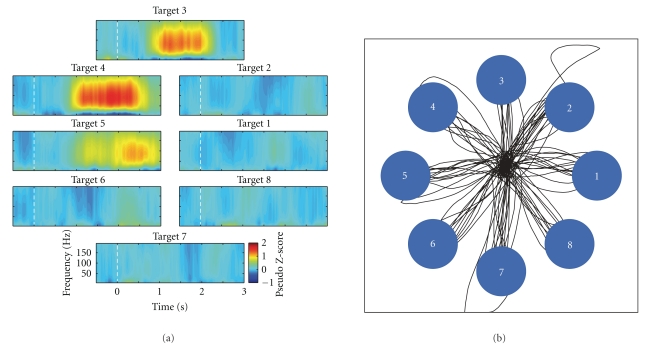
*Closed-loop brain control using simulated ECoG data.* (a) Time-frequency plots of a single simulated ECoG signal averaged across all repetitions of an 8-target center-out cursor control task. Plots are aligned to target presentation at time *t* = 0 (dashed white line). In all, a total of 32 channels of simulated directionally tuned ECoG signals were generated. (b) Real-time cursor trajectories controlled by simulated ECoG signals using the population vector algorithm.

It is worth noting that all the above procedures are conducted in a continuous BMI session without stopping and restarting the Craniux system. This streamlined workflow allows BMI studies to be conducted smoothly and efficiently. Furthermore, steps (2) and (3) can be conducted at any time during a BMI session in parallel with step (4). This allows the neural decoder to be recalibrated on the fly to adapt to any potential changes or nonstationarities of input neural signals, a key element for achieving and maintaining reliable brain control [28].  Figure 5 shows an example of directionally modulated normalized time-frequency data for one ECoG signal saved by the Craniux system, as well as trajectories of the cursor during real-time brain control.

### 3.2. Brain-Controlled Cursor Movement Using Real ECoG Signals Recorded from a Human Subject

**Figure 6:**
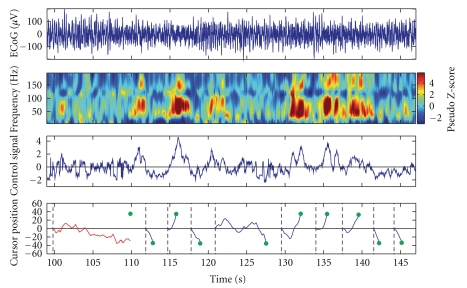
*Closed loop ECoG-based computer cursor control*. One-dimensional computer cursor control using the Craniux system in a subject implanted with ECoG electrodes. Top. Raw time-domain ECoG signal for one of two electrodes used for cursor control. Top-middle. Time frequency data saved by the Craniux system for the same electrode. Bottom-middle. Control signal generated by the Craniux system to control computer cursor movement. Positive control signal values move the cursor in the up direction, while negative control signal values will move the computer cursor down. Note that control signal values are unitless as they have been normalized to zero-mean and unit variance. Bottom. Vertical cursor positions generated by the neural control signal. Dashed black lines represent target onset, green circles indicate the position of presented targets, blue lines indicate cursor trajectories for successful trials, and red lines indicate cursor trajectories for unsuccessful trials.

 Further validation of the Craniux system was conducted in a human subject undergoing subdural epilepsy monitoring. Informed consent was obtained from the subject prior to testing; all experimental procedures were approved by the University of Pittsburgh Institutional Review Board and followed all guidelines for human subject research. Experimental methods used were similar to those presented in [29], with the exception that the Craniux system was used for data collection and brain control. Standard ECoG electrodes exhibiting high-gamma band modulation in response to overt movement screening tasks were chosen for use in closed-loop control. High-gamma band power (70–110 Hz) of two neighboring ECoG electrodes was used to control the vertical movement of a cursor with a push-pull scheme, with the cursor control signal calculated according to:

(4) $$\begin{array}{c} c_{y}=a\left(s_{1}-s_{2}\right)-b, \end{array}$$

where *c*_*y*_ is the one-dimensional control signal, *s*_1_ and *s*_2_ are the high-gamma band power of the two neighboring electrodes used for control, and *a* and *b* are gain and offset terms used to normalize the control signal to zero mean and unit variance. Thus, in order to achieve satisfactory brain control, the subject had to decorrelate the activity of the two electrodes to generate the desired cursor control signal. Brain control sessions began with the collection of baseline data for normalization purposes as described in the previous section. Individual trials began with the placement of the cursor at the center of the computer screen along with the presentation of one of two peripheral targets located in the vertical plane of the workspace (e.g., a “center-out” task). Trials in which the subject was able to hit the presented target within the maximum trial length of 10 seconds were deemed successful; failure to do so resulted in an unsuccessful trial. All trials were followed by an inter trial interval of 2 seconds in which neither the cursor nor the target were visible.  Figure 6 shows the results of one brain control session, during which the subject was able to achieve an 88% success rate.

### 3.3. System Timing

 To evaluate the consistency of system performance, timing characteristics were analyzed for a typical Craniux setup using 15th order autoregressive (AR) spectral estimation of 10 Hz frequency bins over 0.5 second windows of simulated neural data (see Section 3.1), the linear decoder signal processing engine, and the center-out cursor control application engine. Analog and digital data were sampled by the g.USBamp amplification system at 1200 Hz and acquired directly by Craniux at a 33.3 ms frame rate. To trigger timing events, a digital signal was sent from Craniux back to the digital input of the amplifiers, so that the timing events could be acquired precisely and synchronously with the raw analog input signal at 1200 Hz.

Three different timing tests were conducted: a system processing test, a display update rate test, and an overall system latency test. The first two tests (system processing and display update rate) were performed on both a single computer (Windows XP x86 operating system, Intel Core i7 CPU 920 @ 2.67 GHz, 2.49 GB RAM, 2 NVIDIA GeForce 9800 GT video cards) and with Craniux distributed across the network, so that data acquisition, spectral estimation, and GUIs were hosted on one computer (the same as that used for local timing test, see above) while signal processing and Application engines, including the 3D render window, were hosted on a separate computer (Windows XP x86 operating system, AMD Athlon 64 FX-62 Dual Core CPU @ 2.81 GHz, 3.5 GB RAM, NVIDIA GeForce 7900 GS video card). For both configurations, tests were conducted using 16, 32, and 64 channels of data. The third test (system latency) was run only on the single-computer configuration with processing performed on 32 channels of data.

The first test used 5,000 consecutive frames of collected data to measure the system processing time, the time between the arrival of a block of data from the amplifier, and the time when the Craniux system had finished all processing on the data and begun waiting on the next block. These results are shown in the second column of Table 2. As expected, processing time was found to increase with the number of processed channels but remained below the 33.3 ms time required to maintain a consistent frame rate and prevent the loss of data. Distributing Craniux across the network showed improvements in processing time for all channel configurations. Since processing time is only required to remain below the frame rate, running Craniux as a distributed system is not necessary unless the system is under a heavy load. AR spectral estimation was found to require the most processing time, especially as the number of channels increased. These results indicate the extra processing time made available when Craniux is run as a distributed system could easily be utilized to run more complex signal processing algorithms or to decrease the frame rate.

The second test also used 5,000 consecutive frames of data but now measured the refresh rate, the amount of time between consecutive display updates on the center-out cursor control engine. The results are shown in the third column of Table 2. The refresh time was found to be 33.3 ms for all configurations, precisely what would be expected given the system frame rate. Furthermore, the low variability of this timing indicates that the user would experience a consistent cursor update with no noticeable jitter. 

The final test measured system latency, the elapsed time between a neural signal event, and the point in time when the Craniux system can generate an action in response to this event. A 10 Hz sine wave with zero offset was input to 1 channel of the amplifier; this channel of data was fed through the Craniux system to the point at which the display was updated in the center-out cursor control engine, occurring just before processing fully completes and the system begins waiting on the next data block. At this point, if a zero crossing was detected on the sine wave, the digital output bit being written back to the amplifier was flipped. In this case, the elapsed time between a zero crossing of the sine wave (a simulated neural event) and the bit value change (the time of the system response) indicates the system latency. Data was collected for 5,000 consecutive sine wave zero-crossings, with zero crossing events symmetrically distributed about the center of each 33.3 ms data frame. In distributing the zero crossings in this way, it is known that the latency should have an average of slightly less than half the frame length plus the mean processing time (33.9 ms for this configuration) and a range nearly equal to the frame length. The latency was found to have a mean and standard deviation of 33.2 ± 9.6 ms, meeting all expectations.

## 4. Discussion

 Craniux is a powerful, yet simple and easily extendable, open-source framework for BMI studies that require high-performance real-time BMI software. Currently, a number of open-source software solutions for BMI research are available for academic use. These software packages include extremely specialized, high-overhead systems used in nonhuman primate BMI research [8, 28, 30], highly-modular, visual-programming-based software platforms such as OpenViBE [31], as well as portable, lightweight systems for human BMI research [13]. Software tools for more specific BMI research applications have also been made available, from toolboxes allowing for the interfacing of MEG systems in real time for BMI use [32, 33] to real-time brain mapping software capable of quickly identifying signals from electrocorticographic electrodes related to cortical activity corresponding to overt movement, speech, and sensory stimulation [34, 35].

The Craniux framework is inspired by the system architecture design of BCI2000, and we believe that it takes advantage of several unique features of the LabVIEW graphical development environment for developing real-time BMI software. By making it an open-access and open-source software framework, we hope to serve the research community on at least two fronts. First, at the basic level, Craniux is a BMI software solution with an easy-to-use graphical user interface. Those researchers interested in BMIs can use this software to conduct research without writing custom software. Second and most importantly, we hope this framework will facilitate the development of new BMI paradigms and signal processing algorithms by the research community through providing the basic functionalities of BMI system operation, allowing researchers to focus on the development of their specific research questions. Finally, as this framework is set up in the LabVIEW environment, it naturally inherits the many advantages offered by the high-level graphical nature of LabVIEW programming.

In its current form, the Craniux framework demonstrates the benefits and ease with which it can be used and modified to develop new BMI paradigms and algorithms. The simplicity of the LabVIEW programming language makes the creation of new BMI engines accessible to individuals who may not be familiar with object-oriented programming. Would-be developers can simply take one of the provided engine templates, implement their desired operation, and save the engine under a new name (this process is described in greater detail in the supplemental materials). This new engine will then be available for use in the Craniux framework, without the need for the compiling of code down to executables as required by programming languages such as C/C++. The debugging of newly created engines can also be easily performed during run time through the use of LabVIEW's built-in debugging tools. The dataflow-driven nature of Craniux further simplifies debugging, allowing system execution to be halted and resumed at any point during operation without the loss of the current state of the system. These tools, along with advanced data visualization options, make the rapid prototyping of highly sophisticated neural signal processing techniques possible.

Craniux currently offers a rich set of options to visualize BMI data on the fly at multiple processing stages in various formats. Neural signals, such as EEG, MEG, or ECoG, can be viewed as scrolling time-frequency plots or dynamic spatiotemporal plots in the frequency domain. This is beneficial for online examination of neural signal quality, as certain features may be difficult to view in a simple plot of time-domain raw neural signals. The results of calculations performed during the training and application of neural decoding algorithms can also be visualized on the fly, providing researchers with the opportunity to select neural signal features, visualize decoding weights, and examine decoder outputs without suspending operation of the system. For example, our implementation of the population vector algorithm allows researchers to dynamically change the value of the *R*-squared threshold used for feature selection, view the preferred direction distribution of the currently selected features, and view the instantaneous contribution of all features to the control signal output by the algorithm. This visualization capability is of particular importance when using and developing sophisticated decoding algorithms, as it allows BMI researchers to judge the validity of the decoding weights on the fly and make adjustments of neural signal processing and other BMI experiment parameters accordingly.

We have also shown the potential for enhancement in Craniux performance through its distribution across multiple network hosts, as assigning individual engines to separate computers eliminates the possibility of competition between engines for system resources. The separation of graphical user interface elements from real-time engines further improves system performance by ensuring the real-time engine execution is not affected by user interface interaction events or data visualization. Furthermore, the capability to distribute the Craniux system across multiple network hosts could prove especially useful in long-term human BMI studies. Experimental sessions could be run remotely on a daily basis, eliminating the need for either subjects or investigators to travel to participate in these sessions. This will become important as BMI technology moves into preclinical and clinical trials.

Craniux also offers a streamlined workflow for BMI research. It allows for on-the-fly control of specific experimental parameters, offering experimenters great flexibility for BMI user training. For example, an experimenter can quickly adjust the output gain of a neural decoder if it is deemed that a brain-controlled cursor is moving in the correct direction but with a very low speed. In our experience, this flexibility is critical for effective BMI training. Meanwhile, the Craniux system is capable of capturing all changes in experimental parameters along with BMI data, allowing researchers to perform offline analysis of BMI sessions. Furthermore, various BMI procedures, including the collection of baseline data for the normalization of neural signals and the training of neural decoding algorithms, may be performed without the cessation of system operation. This provides both BMI researchers and experimental subjects with a seamless experience in which system parameters can be continuously updated to improve BMI performance.

It should be noted that the timing of the Craniux system is dependent on the timing of the acquisition engine, which currently can be driven by UDP packets sent from neural acquisition hardware or controlled explicitly by the acquisition engine itself (e.g., the “SimECoG” engine). Any number of neural recording hardware solutions may be used for BMI operation provided that data recorded by these devices can be packaged and transmitted via UDP. Additionally, hardware-specific acquisition engines can also be created within the Craniux framework.

In addition, it is important to mention that editing or developing new BMI engines in the Craniux framework requires the purchase of LabVIEW. However, it is not uncommon for open-source research tools to be built upon commercial software; two such examples are the EEGLAB [36] and FieldTrip packages for neural data analysis. These packages are both built upon MATLAB, an extremely powerful commercial data analysis software package. Just as many researchers are now using MATLAB instead of custom-written C programs for data analysis, we believe that the time and effort saved by the use of the Craniux system in BMI software development will outweigh the cost of the LabVIEW software. Furthermore, if the Craniux software is to be used as a self-contained out-of-the-box software package, all engines can be compiled down to binary executable files and run using the freely available LabVIEW runtime engine, eliminating the need for the LabVIEW software. Finally, as demonstrated in Section 3, given the computing capability of current personal computers and the code optimization inherently performed by the LabVIEW environment, the overall performance of the Craniux system is comparable to BMI systems developed using other programming languages. Hence, the gain from using the high-level LabVIEW programming environment does not come at the expense of significant sacrifices in system performance.

The Craniux software package, including in-depth documentation and detailed operation instructions for all engines, has been made available free of charge to academic institutions and can be accessed at http://hrnel.pitt.edu/Software.html. The Craniux software package can be downloaded as a library of LabVIEW virtual instruments (VIs), and all stable system updates will be made available for download.

## 5. Conclusion

 While other open-access open-source BMI software solutions are currently available, we feel that the Craniux software package fills a specific need in the realm of BMI research. Powerful yet lightweight, this system allows experimenters to rapidly develop and test cutting-edge technology in an online environment, whether it is new neural signal processing techniques, new neural decoders, or advanced prosthetic devices. This system offers an easy-to-use “out-of-the-box” solution for BMI research as well as other neural data visualization and processing purposes. Additionally, the Craniux system provides an extendable framework through the provision of template engines. The provided framework possesses the basic fundamental architecture for running closed-loop BMI experiments and enables other researchers to take advantage of LabVIEW functionality to design and conduct novel experimental paradigms without the need to implement their own core system framework. It is also worth noting that functionality offered by the Craniux framework also lends itself useful for other neuroscience research and even neurorehabilitation applications that could benefit from real-time processing and visualization of neural data, such as cortical source imaging using EEG or MEG recordings. It is with these characteristics in mind that we feel the Craniux software package will prove an important addition to the BMI research community.

## Supplementary Material

Supplemental material included here provides detailed information on software requirements, startup instructions, operation, and engine creation for the Craniux software system.

## Figures and Tables

**Table 1:** List of current acquisition, signal processing, and application engines.

Engine name	Engine type	Description
Acquisition template	Acquisition	“Empty” acquisition engine generating random data used to maintain system dataflow
Read UDP binary	Acquisition	Reads raw neural data transmitted via UDP
SimECoG	Acquisition	Generates synthetic ECoG data

Signal processing template	Signal processing	“Empty” signal processing engine used to maintain system dataflow
Linear decoder	Signal processing	Generates a control signal using linear combinations of input features
Population vector	Signal processing	Generates a control signal using the population vector algorithm [18]
OLE	Signal processing	Generates a control signal using the optimal linear estimator algorithm [19]

Application template	Application	“Empty” application engine used to maintain system dataflow
Center-out cursor control	Application	Two or three-dimensional cursor control application
Threshold Crossing	Application	Sends UDP commands to an external device when control signals cross user-defined thresholds
Circle drawing	Application	Circle/ellipse-drawing application [20]
Biofeedback	Application	Displays real-time feedback of a neural control signal to the subject

**Table 2:** *Characterization of system timing.* Craniux system processing time, refresh rate, and latency for local and network system configurations under various processing loads. Values shown are mean timing values plus or minus one standard deviation from the mean.

System configuration	Processing time (ms)	Refresh rate (ms)	Latency (ms)
Local, 16 channels	12.8 ± 0.7	33.3 ± 0.5	N/A
Network, 16 channels	9.9 ± 0.6	33.3 ± 0.5	N/A
Local, 32 channels	17.8 ± 0.8	33.3 ± 0.7	33.2 ± 9.6
Network, 32 channels	15.2 ± 1.0	33.3 ± 0.4	N/A
Local, 64 channels	28.0 ± 1.3	33.3 ± 0.7	N/A
Network, 64 channels	24.9 ± 0.9	33.3 ± 0.4	N/A

## References

[1] Schwartz AB, Cui XT, Weber D, Moran DW (2006). Brain-controlled interfaces: movement restoration with neural prosthetics. *Neuron*.

[2] Wang W, Collinger JL, Perez MA (2010). Neural interface technology for rehabilitation: exploiting and promoting neuroplasticity. *Physical Medicine and Rehabilitation Clinics of North America*.

[3] McFarland DJ, Sarnacki WA, Wolpaw JR (2010). Electroencephalographic (EEG) control of three-dimensional movement. *Journal of Neural Engineering*.

[4] Mellinger J, Schalk G, Braun C (2007). An MEG-based brain-computer interface (BCI). *NeuroImage*.

[5] Schalk G, Miller KJ, Anderson NR (2008). Two-dimensional movement control using electrocorticographic signals in humans. *Journal of Neural Engineering*.

[6] Heldman DA, Wang W, Chan SS, Moran DW (2006). Local field potential spectral tuning in motor cortex during reaching. *IEEE Transactions on Neural Systems and Rehabilitation Engineering*.

[7] Hochberg LR, Serruya MD, Friehs GM (2006). Neuronal ensemble control of prosthetic devices by a human with tetraplegia. *Nature*.

[8] Velliste M, Perel S, Spalding MC, Whitford AS, Schwartz AB (2008). Cortical control of a prosthetic arm for self-feeding. *Nature*.

[9] Ganguly K, Carmena JM (2009). Emergence of a stable cortical map for neuroprosthetic control. *PLoS Biology*.

[10] Koyama S, Chase SM, Whitford AS, Velliste M, Schwartz AB, Kass RE (2010). Comparison of brain-computer interface decoding algorithms in open-loop and closed-loop control. *Journal of Computational Neuroscience*.

[11] Adee S Dean Kamen's `luke arm' prosthesis readies for clinical trials. http://spectrum.ieee.org/biomedical/bionics/dean-kamens-luke-arm-prosthesis-readies-for-clinical-trials.

[12] Adee S Winner: the revolution will be prosthetized. http://spectrum.ieee.org/robotics/medical-robots/winner-the-revolution-will-be-prosthetized.

[13] Schalk G, McFarland DJ, Hinterberger T, Birbaumer N, Wolpaw JR (2004). BCI2000: a general-purpose brain-computer interface (BCI) system. *IEEE Transactions on Biomedical Engineering*.

[14] Schalk G BCI2000 provided the basis for experiments in the following peer-reviewed journal papers.

[15] Bcpy2000. http://bci2000.org/downloads/BCPy2000/BCPy2000.html.

[16] Wilson JA, Mellinger J, Schalk G, Williams J (2010). A procedure for measuring latencies in brain computer interfaces. *IEEE Transactions on Biomedical Engineering*.

[17] The NI TDMS file format. http://zone.ni.com/devzone/cda/tut/p/id/3727.

[18] Georgopoulos AP, Schwartz AB, Kettner RE (1986). Neuronal population coding on movement direction. *Science*.

[19] Salinas E, Abbott LF (1994). Vector reconstruction from firing rates. *Journal of Computational Neuroscience*.

[20] Schwartz AB, Moran DW, Reina GA (2004). Differential representation of perception and action in the frontal cortex. *Science*.

[21] Nagle J Congestion control in IP/TCP internetworks. http://tools.ietf.org/html/rfc896.

[22] Leuthardt EC, Schalk G, Wolpaw JR, Ojemann JG, Moran DW (2004). A brain-computer interface using electrocorticographic signals in humans. *Journal of Neural Engineering*.

[23] Keshner MS (1982). 1/f NOISE. *Proceedings of the IEEE*.

[24] Tallon-Baudry C, Bertrand O, Hénaff MA, Isnard J, Fischer C (2005). Attention modulates gamma-band oscillations differently in the human lateral occipital cortex and fusiform gyrus. *Cerebral Cortex*.

[25] Edwards E, Nagarajan SS, Dalal SS (2010). Spatiotemporal imaging of cortical activation during verb generation and picture naming. *NeuroImage*.

[26] Schwartz AB, Kettner RE, Georgopoulos AP (1988). Primate motor cortex and free arm movements to visual targets in three-dimensional space. I. Relations between single cell discharge and direction of movement. *Journal of Neuroscience*.

[27] Wang W, Chan SS, Heldman DA, Moran DW (2007). Motor cortical representation of position and velocity during reaching. *Journal of Neurophysiology*.

[28] Taylor DM, Tillery SIH, Schwartz AB (2002). Direct cortical control of 3D neuroprosthetic devices. *Science*.

[29] Wang W, Degenhart AD, Collinger JL Human motor cortical activity recorded with micro-ECoG electrodes during individual finger movements.

[30] Bacher D, McFerron J, Krishnamurthy N, Batista A An experimental rig for closed-loop neuroprosthetics.

[31] Renard Y, Lotte F, Gibert G (2010). OpenViBE: an open-source software platform to design, test, and use brain-computer interfaces in real and virtual environments. *Presence: Teleoperators and Virtual Environments*.

[32] Sudre G, Wang W, Song T rtMEG: a real-time software toolbox for brain-machine interfaces using magnetoencephelography.

[33] The FieldTrip buffer for real-time access to EEG/MEG data. http://fieldtrip.fcdonders.nl/development/realtime/buffer.

[34] Schalk G, Leuthardt EC, Brunner P, Ojemann JG, Gerhardt LA, Wolpaw JR (2008). Real-time detection of event-related brain activity. *NeuroImage*.

[35] Schalk G, Brunner P, Gerhardt LA, Bischof H, Wolpaw JR (2008). Brain-computer interfaces (BCIs): detection instead of classification. *Journal of Neuroscience Methods*.

[36] Delorme A, Makeig S (2004). EEGLAB: an open source toolbox for analysis of single-trial EEG dynamics including independent component analysis. *Journal of Neuroscience Methods*.

