# Supplementary material for: Craniux: A LabVIEW-Based Modular Software Framework for Brain-Machine Interface Research
Source: Comput Intell Neurosci. 2011 Apr 7;2011:363565. doi: 10.1155/2011/363565 (PMC3113411; doi:10.1155/2011/363565)
Supplement: Supplementary file 2 [file 363565.f2.pdf]

Craniux: A LabVIEW-based Modular Framework for Brain-Machine Interface Research
Supplemental Material

LabVIEW requirements

Craniux has been developed and fully tested using the LabVIEW 2010 Professional Develop System (version 10.0). The Advanced Signal Processing Toolkit is currently required for operation. In particular, this toolkit is necessary in order to perform autoregressive spectral estimation using the 'TSA AR Spectrum' virtual instrument (VI); this requirement will be removed in a future release of Craniux. Craniux cannot currently be run using the LabVIEW 2010 'Student' or 'Basic' Editions, as these versions of LabVIEW do not by default include the required Advanced Signal Processing Toolkit. In order to run Craniux using these versions of LabVIEW, the missing spectral estimation VI must first be replaced by a new VI implementing the user's desired spectral estimation method. Finally, a fully-functional version of Craniux has also been made available for the LabVIEW 2009 Professional Development System (version 9.0), though this version also requires the Advanced Signal Processing Toolkit.

Startup instructions

Installation of Craniux is performed with a standard installation package. When complete, the Windows Start Menu will contain an entry for Craniux, and within that, the command "Craniux", which will launch the system. (Alternatively, a user can double-click the file "Craniux Launcher.vi" in the directory where the system was installed.)

Launching the system brings up the main window, referred to as the "Craniux Launcher". Running a Craniux configuration involves three steps:

1. *Selecting appropriate engines*. These fields are populated with default values, and can be changed prior to loading engines, or when the system is subsequently suspended. Optionally, different hosts can be selected for running the engines.
2. *Loading the engines*. This is performed by pressing the "Load Modules" button, which performs initialization steps for the engines.
3. *Running the engines*. Once the engines are selected and loaded, the "Start" button will become available. Clicking this will start the data flow process through the engines.

Once the engines are running, they can be temporarily paused by clicking the "Suspend" button. While suspended, engines can be switched out but hosts cannot be changed. To stop Craniux completely, click the "Stop" button.

Enforcement of deterministic engine execution

In general, there is little ambiguity in the order of execution of core operations as the data-driven nature of LabVIEW programming dictates that execution follows the availability of data. Furthermore, if more explicit control of execution is desired, a number of LabVIEW components can be used to accomplish this. During the development of Craniux software, we have taken advantage of LabVIEW's standard mechanisms to ensure deterministic execution through the use of sequence structures, queues, and other synchronization elements. These actions are explained in detail below.

Main Sequence Execution

Deterministic execution of the main sequence loop in an engine is driven by incoming TCP data or the availability of hardware-acquired data in conjunction with a sequence structure. In the first frame of the structure, engine execution is halted until an incoming packet of data is received from the previous engine. Once this data is parsed and written to local controls (i.e. a variable in LabVIEW with an associated GUI element on the VI front panel) within the engine, execution proceeds to the next frame in the sequence. It is here that engine-specific implementation code is to be placed. Within this sequence frame the automatic parallelism inherent to LabVIEW is the most useful, as the execution of the current engine, and subsequently the execution of all other engines, is not allowed to proceed until all operations present in this sequence frame are complete. Once this occurs, execution proceeds to a subsequent

sequence frame, where data to be transmitted to the next engine is packaged up and transmitted via TCP. Additionally, transmission of data to be saved is transmitted to the data saving manager and visualization data to the engine's associated GUI occurs within this frame. In short, this execution can be summarized in the following *sequential* steps, with parallel dataflow-driven execution occurring within each step:

1. Wait for incoming TCP data
2. Perform some engine-specific operation
3. Package up and send outgoing TCP data, save data, and send visualization data to GUI.

Updating of Parameters by GUIs

Parameter updates are triggered by user input on the front panel of an engine's GUI. Value change events for all GUI parameters are monitored within the GUI; updated parameter values are packaged up and sent via TCP to the engine as soon as a value change event is triggered. Within the associated engine, a TCP listener waits for incoming parameter updates in a process operating in parallel to that of the main sequence loop (see above). Once a parameter update packet is received, this packet is placed into a queue that is read immediately following transmission of data to the next engine (step '3', described in the preceding section). A semaphore keeps parameter values from updating during execution of the 'engine-specific operation' frame (step '2', above). This ensures that instances where parameter values are read multiple times within the engine-specific operation frame use the same parameter value, eliminating race conditions.

Sending of Visualization Data

Sending of visualization data occurs during sequence frame (3) identified above. Here, the values of all variables to be transmitted to the GUI for visualization are read and packaged into a single TCP packet. This packet is placed into a single-element queue retaining the most recent packet (a 'lossy' queue), which is read by a sending process running in parallel to execution of the main sequence. This packet is read from the queue when it becomes available and then sent via TCP to the user interface. In instances where the transfer of a packet is not performed before the next packet is available, the previous packet is overwritten by the new packet, ensuring only the most recent visualization data is sent to the GUI. The user interface then parses these packets and writes visualization data to the appropriate GUI variables. From here, raw visualization data is processed as needed and updated on the GUI.

Data Saving

Data is initially saved in LabVIEW's TDMS format. To specify what data is saved, the developer must create two string arrays in each engine's initialization frame. The first string array specifies the names of 'sampled variables' that should be saved for every sample of neural data that is processed. The second array is names of 'saved controls,' which are engine parameters that should only be saved when their value is changed during run-time. The developer also has the option of automatically including all variables that aren't in the sampled variables list as saved controls. During initialization, arrays of references to these two sets of variables are created. A reference to the data packet number is included in both groups so that the experiment can be reconstructed afterwards with the data properly aligned in time.

The values of sampled variables are placed in a queue after the engine completes its main execution frame. The values of saved controls are placed in the queue after one of them is changed. In parallel to the main execution loop, values are pulled from the buffer and sent via the TCP framework to a data saving manager on the user interface host. This module formats and streams the data to the TDMS file. Each channel of streamed data is notated by the variable name, the engine it came from, and whether it is a sampled variable or saved control. Array size information is also saved to ensure correct reconstruction of each sample from the streams of data. A single TDMS file is saved for each experimental run; stopping or suspending system execution closes all references to the current data file.

A separate LabVIEW VI has been created to convert Craniux TDMS files into the MATLAB (Mathworks, Inc.) MAT format. These MAT files contain a data structure with 6 sub-structures: one for each engine type (signal source, signal processing, or application) paired with each data type (sampled variables and controls). Each channel of scalar data in the TDMS file is converted to an array, with the data packet number providing the time index for each element, and placed in the proper sub-structure. Each channel of array data is parsed using the corresponding size data. Since the size of the array could be dynamic, the data is placed in a cell array with each cell corresponding to a sample of the array for each data packet number. Currently only 1D and 2D arrays can be saved in this way.

Transfer of information between engines

As mentioned in the manuscript, transfer of information between engines occurs via TCP. Names of variables to be transmitted from one engine to another are defined in a string array located in an engine's initialization procedure. Based on these names, an array of references to the desired variables is generated; this array is used when sending data from one engine to the next. Within the third sequence frame of the main sequence defined previously (see 'Main Sequence Execution'), this array of references is used to obtain the current values of the specified variables to be transmitted. Two arrays are then generated: an array of strings representing the names of the variables being transmitted, and an array of type 'variant' data containing the value of each of these variables. These two arrays are then packaged into a single TCP packet and transmitted to the next engine in the loop.

This transmitted packet is then read and reconstructed into a pair of name/value arrays in the same format as sent from the previous engine. Then, for each variable name, the corresponding variable value is written to the variable on the current engine that has the same name and data type. If no such variable is found, no action is taken by the system. In this manner, a developer must ensure that transmitted variables are consistent between engines.

When receiving signals via UDP from external hardware the Craniux system will wait indefinitely for the next packet of data. Currently, there is no timeout check for incoming data, though this could easily be added to the software. Parallel processing is used to ensure incoming data is continuously saved to disk without loss. In this manner, the real-time processing chain is always operating with the most recent block of data.

Creation of new engines

The common programming work required for the creation of new engines has already been implemented in 'template' engines and GUIs for each module type. Though detailed steps for the creation of new engines from the provided templates are provided in the documentation, an abbreviated version is provided below.

1. *Implementation of engine operation*. When implementing a new engine, the primary task is to construct engine code that will be executed during each iteration of data processing in the Craniux system. The function of this code is to use the current iteration's data received from the previous engine, process it according to whatever algorithm is being implemented, and pass results on to the next engine. A specific space has been reserved in the template engines (marked with the comment '<main program here>') for building this code. In addition to the data received in the processing chain, an engine may also rely on user input or other sources of data, which can be stored from iteration to iteration and/or saved to disk.

2. *Specification of transmitted and sample variables*. The second task is to indicate, via simple lists of names, four sets of variables:
    a. Data to be transmitted to the next engine.
    b. Variables that will be saved every for every iteration through the engine.
    c. Variables that are saved only when modified by the user (via the engine's GUI).

d. Variables or controls that are sent to the user interface module (the GUI) for visualization.

3. *Creation of the user interface*. The last step for creating a working engine is adapting the GUI template to the engine's requirements. In its simplest form, this consists of placing controls in the GUI's main window, and then constructing a list of these controls that should be transmitted to the engine when changed. Visualization of processing can also be added in the form of LabVIEW's many charting and graphing options.
